# Rigorous mathematical optimization of synthetic hepatic vascular trees

**DOI:** 10.1098/rsif.2022.0087

**Published:** 2022-06-15

**Authors:** Etienne Jessen, Marc C. Steinbach, Charlotte Debbaut, Dominik Schillinger

**Affiliations:** ^1^ Institute of Mechanics, Computational Mechanics Group, Technical University of Darmstadt, 64287 Darmstadt, Germany; ^2^ Institute of Applied Mathematics, Leibniz Universität Hannover, 30167 Hannover, Germany; ^3^ IBiTech – Biommeda, Ghent University, Ghent, Belgium

**Keywords:** synthetic vascular trees, rigorous geometry optimization, nonlinear optimization problem, heuristic topology optimization, liver corrosion cast, validation

## Abstract

In this paper, we introduce a new framework for generating synthetic vascular trees, based on rigorous model-based mathematical optimization. Our main contribution is the reformulation of finding the optimal global tree geometry into a nonlinear optimization problem (NLP). This rigorous mathematical formulation accommodates efficient solution algorithms such as the interior point method and allows us to easily change boundary conditions and constraints applied to the tree. Moreover, it creates trifurcations in addition to bifurcations. A second contribution is the addition of an optimization stage for the tree topology. Here, we combine constrained constructive optimization (CCO) with a heuristic approach to search among possible tree topologies. We combine the NLP formulation and the topology optimization into a single algorithmic approach. Finally, we attempt the validation of our new model-based optimization framework using a detailed corrosion cast of a human liver, which allows a quantitative comparison of the synthetic tree structure with the tree structure determined experimentally down to the fifth generation. The results show that our new framework is capable of generating asymmetric synthetic trees that match the available physiological corrosion cast data better than trees generated by the standard CCO approach.

## 1. Introduction

**Figure 1.**
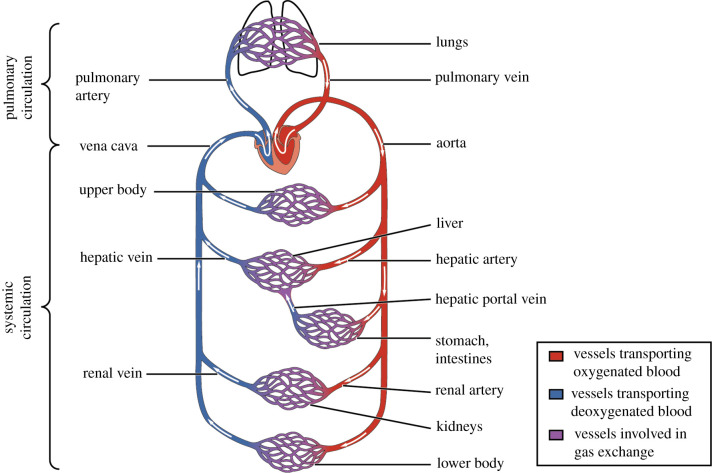
Schematic overview of the cardiovascular system [2].

The cardiovascular system of the human body supplies the cells with vital nutrients by permitting blood to circulate throughout the body [1]. The heart pumps the blood through vessels, categorized into arteries (transporting blood away from the heart) and veins (transporting blood towards the heart). The cardiovascular system is further divided into the pulmonary circulation and the systemic circulation. In the pulmonary circulation, deoxygenated blood is carried from the heart to the lungs, and oxygenated blood returns to the heart. By contrast, the systemic circulation carries oxygenated blood from the heart to the rest of the body, reaching the other organs. The blood enters these organs through different branches of the aorta, where arteries distribute it. The arteries split into smaller and smaller arteries until they reach the arterioles, which are the last arterial branches prior to entering the microcirculation. After the blood is distributed at the microcirculatory level and interacts with the organ’s cells, the capillaries merge to bring the deoxygenated blood back through the venules, which merge into veins. Finally, the blood leaves the systemic circulation through either the superior or inferior vena cava back to the heart. The complete cardiovascular system is schematically shown in figure 1.

Formally, the systemic circulation can be divided into two functional parts: macrocirculation and microcirculation. In the microcirculation, nutrients and oxygen diffuse towards the organ’s cells. Here, the main functions of the different organs are carried out, e.g. synthesizing proteins and detoxification in the liver. By contrast, macrocirculation mainly distributes oxygenated blood evenly throughout the organs and then recollects the deoxygenated blood. The task of distributing and collecting blood leads to specific branching patterns inside the organs. These sets of branches, at least one for arteries and one for veins inside each organ, are called vascular trees. The general structure of a vascular tree mainly depends on the organ supplied, with the main factors being the organ’s shape, the amount of blood supply and the microcirculation structure. Furthermore, a distinction between solid organs (such as the liver) and hollow organs (such as the stomach) must be made.

In general, vascular trees are patient specific, and clinicians cannot derive them from statistical measures alone. Having detailed patient-specific data on vascular trees is essential to help further improve many clinical treatment strategies, for example determining suitable cut patterns in liver resection or optimizing targeted chemotherapy for cancer patients. An essential tool for obtaining patient-specific data on vascular trees *in vivo* is non-invasive medical imaging such as computed tomography (CT) or magnetic resonance imaging (MRI). Their maximum resolution for *in vivo* imaging, however, even with the advances made in the last decade, is still limited. Therefore, to understand vascular trees down to the arterioles and venules, *ex vivo* methods must be used, but these are often time-consuming and require specialized equipment, making them expensive. Examples are cryomicrotomes in human hearts [3] or corrosion casting of the liver [4]. The latter uses curable resin and a maceration process to extract a cast of the blood vessels from the organ.

An alternative approach is based on the synthetic generation of vascular trees with the help of a computer. Starting from available low-resolution patient-specific imaging data, synthetic vascular trees can potentially fill in the missing data to obtain a high-resolution model-based representation of the hierarchical vascular system. These synthetic vascular trees are based on optimality principles whose goal is to minimize the metabolic cost [5–7]. The assumption is that the individual branchings, defining the structure of the tree on the macroscale, form under these principles. Most existing methods generate vascular trees based on these optimization principles and assume that flow is distributed evenly into a pre-defined perfusion volume. Such synthetic trees can be generated for any pre-arteriolar refinement level. A number of different methods exist that differ in terms of the optimization algorithms and the constraints for guiding the optimization.

The most well-known approach for generating vascular trees is the constrained constructive optimization (CCO) method, first proposed by Schreiner & Buxbaum [8] and later extended to three-dimensional non-convex domains [9]. It is based on modelling blood flow using Poiseuille’s law and optimizing bifurcations using Murray’s law [10]. The underlying algorithm starts from an initial vascular structure and iteratively adds new segments while optimizing the local tree structure (topology and geometry) at each bifurcation. Since CCO plays a central role in the topology optimization of our framework, we review the method in more detail later. CCO can reproduce a qualitatively reasonable distribution of segments, but fails to capture the asymmetric branching patterns that characterize most real vascular trees. Several adaptations to CCO have been proposed that attempt to remove this limitation, for example using new constraints or new intermediate processing steps for generating organ-specific vascular systems [11–13]. Moreover, owing to the sampling of new segments the results of CCO-generated trees are largely dependent on random seeds [14]. In Hahn *et al.* [15], an alternative method known as global constructive optimization (GCO) was introduced. It starts by defining random points inside the perfusion volume. These points are kept fixed throughout the optimization and are the leaf nodes of the resulting vascular tree. The goal is now to construct the topology and positions of the internal nodes of the vascular tree. Optimization is driven by successively connecting all leaf nodes to existing internal nodes (starting with only the root node) and then using splitting and pruning steps to create new internal nodes. Thus the method iterates through suboptimal global structures until the tree reaches a suitable level of refinement. Organ-specific methods for generating vascular trees have also been introduced, e.g. for the stomach [11] and the liver [13,16,17].

A recent method, proposed by Keelan *et al.* [18], is based on the assumption that the limitations in the results of CCO and its variants are caused by the fact that only optima of the local tree structure are explored. Instead of adding intermediate or post-processing steps to CCO, a new approach based on simulated annealing (SA) was introduced to search for the optimum of the global tree structure. Like GCO, this approach generates the leaf nodes beforehand. The optimization step consists in adjusting the topology and geometry of the vascular tree iteratively. It was claimed that the approach will converge against the global minimum if the number of iterations goes to infinity. Results also show a visual convergence of trees with different initial structures to very similar global structures after optimization, a feature no other introduced method was able to reproduce. However, as SA is used for both topology and geometry optimization, the algorithm is extremely costly for decently sized three-dimensional vascular trees and global convergence cannot be guaranteed.

In this paper, we introduce a new framework for generating synthetic vascular trees, which rigorously mitigates the limitations of the CCO approach, achieving results similar to the SA-based method but at a significantly lower computational cost. We start by casting the problem of finding the optimal global tree geometry into a nonlinear optimization problem (NLP). We then specialize the global model for optimizing the local geometry of a single new branching. This rigorous mathematical formulation accommodates efficient solution algorithms and makes changes in boundary conditions and constraints trivial. The framework also includes a discrete optimization step for iterating between different topologies. To this end, it combines CCO with a heuristic subtree-swapping step motivated by the SA approach [18]. We combine the geometry and topology optimization steps into a single algorithmic approach. Unlike the standard CCO approach and its variants, we reduce the resulting volume of the tree significantly and limit the influence of random samples on the final global tree structure. Based on the formal separation of topology and geometry optimization, the efficiency of the algorithm is significantly improved compared with the SA approach. The new framework allows us to generate a synthetic tree inside a non-convex organ up to the pre-arteriolar level, where the microcirculation starts and the tree transmutes into a meshed network of micro-vessels.

## 2. Methods

### 2.1. Model assumptions

We model the vascular tree as a branching network T=(V,A), consisting of nodes u∈V and segments a∈A. The segments are assumed to be rigid and straight cylindrical tubes, and each segment a=uv is defined by its radius $r_{a}$ and the geometric locations of its proximal node $x_{u}$ and distal node $x_{v}$, yielding the length $\ell_{a}=\|x_{u}-x_{v}\|$. The goal is to generate the vascular tree inside a given (non-convex) perfusion volume $\Omega \subset R^{3}$, while homogeneously distributing all terminal nodes (*leaves*) v∈L. The network is perfused at steady state by blood, starting at the feeding artery (*root segment*) down to the leaves at the *terminal segments*. In a real vascular system, the tree transmutes into an arcade network of micro-vessels [19] (mathematically a general meshed graph with cycles) when reaching the arteriolar level (radii in the range 0.02–0.1 mm). As such, the pre-arteriolar level marks a conceptual cut-point of this model since the underlying assumptions are no longer justified [8]. To simplify the model, blood is assumed to be an incompressible, homogeneous Newtonian fluid. Further assuming laminar flow, we can express the hydrodynamic resistance $R_{a}$ of segment a by Poiseuille’s law as

2.1 $$R_{a}=\frac{8\eta }{\pi }\frac{\ell_{a}}{r_{a}^{4}}\;\forall \;a\in A,$$

where η denotes the dynamic viscosity of blood which is assumed constant with *η* = 3.6 cP. We note, however, that the typical radius of the smallest arteries in the pre-arteriolar level is in the range 0.1–0.2 mm, and the so-called Fåhræus–Lindqvist effect [20] should be taken into account for these vessels with

2.2 $$\begin{array}{rl} \eta (r_{a})= & \;1.125(\kappa +\kappa^{2}[6\exp(-170r_{a}/\text{mm})-2.44\exp(-8.09\\ & {\left(r_{a}/\text{mm}\right)}^{0.64})+2.2]) \end{array}$$

and

2.3 $$\kappa =\frac{r_{a}^{2}}{{\left(r_{a}-0.00055\;\text{mm}\right)}^{2}}.$$

This effect describes the change of the blood viscosity based on the vessel diameter and, in particular, the decrease of viscosity as the vessel diameter decreases. This stems from the fact that in smaller vessels the blood cells tend to be in the centre, forcing plasma towards the walls, which decreases the peripheral friction. The pressure drop $\Delta p_{a}$ over segment a can now be computed by

2.4 $$\Delta p_{a}=R_{a}Q_{a}\;\forall \;a\in A,$$

where $Q_{a}$ is the volumetric blood flow through segment a. At individual branchings, the relationship between a parent segment and its daughter segments obeys the power law

2.5 $$r_{uv}^{\gamma }=\sum_{vw\in A}r_{vw}^{\gamma }\;\forall \;v\in V\setminus L,$$

where γ is the *branching exponent*. It has the value 3.0 in Murray’s law [10], which is shown to yield a balance between minimizing the metabolic cost of maintaining blood and power loss for moving blood [21]. In the literature, γ values from 2.0 to 3.0 are generally considered valid for vascular trees [22–26], with, for example, γ = 2.55 minimizing pulsatile flow [25] and γ = 2.7 minimizing vascular wall material [26]. As noted in Schwen & Preusser [13], a constant value γ might not be very realistic and γ should be considered dependent on the branching generation in future.

In addition to the model assumptions, a set of physiological constraints are needed to construct the vascular tree. As suggested in Schreiner & Buxbaum [8], we assume that the tree minimizes the metabolic cost of maintaining blood inside the tree, which is proportional to the tree’s volume,

2.6 $$f_{T}=\sum_{a\in A}\pi \ell_{a}r_{a}^{2}.$$

We further constrain the tree to have equal pressure $p_{\mathrm{term}}$ at all terminal nodes, which are the entry points into the microcirculatory network. Since the tree induces a given total perfusion $Q_{\mathrm{perf}}$ (at the root node) across an overall pressure drop $\Delta p=p_{\mathrm{perf}}-p_{\mathrm{term}}$, this constraint leads to equal outflow at each terminal node.

### 2.2. Constrained constructive optimization

Before we introduce our framework, we first describe CCO in more detail and illustrate key properties of its results via a representative benchmark example. We note that, for visualizing trees, we employ the software POV-Ray [27], where we represent branching points as spheres and segments as cylinders.

#### 2.2.1. Algorithmic background and key properties

**Figure 2.**
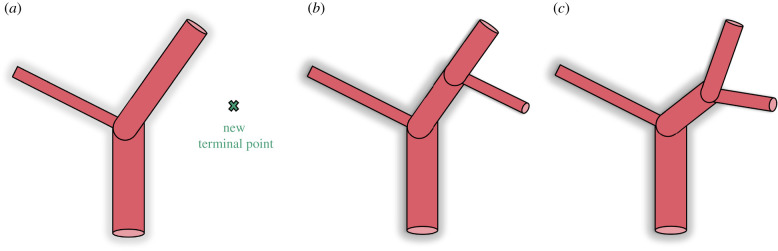
Schematic overview of CCO’s growth algorithm, showing the three main steps. Steps (*b*) and (*c*) are repeated for all neighbouring segments of the new terminal point. (*a*) Sampling of a new terminal point, (*b*) connection to an existing segment and (*c*) optimization of a new bifurcation.

CCO can generate a vascular tree under the assumptions and boundary conditions described above. The main idea behind CCO is to grow the vascular tree incrementally by adding new segments one by one. Each addition consists of three steps. Step 1 is to sample a new terminal point $x_{\mathrm{term}}$ uniformly inside the perfusion volume. The distance of the sampled point to each existing segment must be larger than a pre-defined threshold. This threshold ensures that the new terminal point is compatible with the current tree geometry and leads to a uniform distribution of all terminal points inside the perfusion volume. The distance between the sampled point and a segment is computed by evaluating the orthogonal projection onto the convex line segment. The required threshold is lowered with each iteration to accommodate the growing number of segments inside the perfusion volume. After a new terminal point is found, it is connected to an existing segment in step 2, leading to a new bifurcation. In step 3, the location of this newly created bifurcation is optimized for the lowest total tree volume. Steps 2 and 3 are repeated only for the $N_{\mathrm{con}}$ closest segments of $x_{\mathrm{term}}$, and the connection with the lowest total volume is chosen as permanent. The number $N_{\mathrm{con}}$ of different connections tested will be investigated later. The entire approach is visualized in figure 2. The search for the best connection is an optimization of the local topology, while the search for the best location of the bifurcation point is an optimization of the local geometry of the tree.

The growth algorithm for a tree can run either until a prescribed number of segments are connected or until the radius of new terminal segments is below a certain threshold (usually the minimum radius of the pre-arteriolar level, $r_{\min}=0.1\;\mathrm{mm}$). The main computational burden of CCO is the geometry optimization that follows the introduction of the new bifurcation in each iteration. After a new terminal point is connected to the tree, the constraints and boundary conditions (e.g. equal terminal outflow) do not hold any longer. The hydrodynamic resistance of each segment on the path from the new bifurcation to the root needs to be rescaled to account for this newly created segment, subsequently inducing a rescaling of the root radius. Therefore, all radii need to be recomputed. This rescaling of the tree is a recursive computation starting from the new terminal segment, which is described in detail in Karch *et al.* [9]. Each time the position of a bifurcation is changed, the tree needs to be rescaled in such a manner.

Synthetic trees generated by the CCO approach show good visual agreement with morphological data and have comparable mean radii over all generations. However, one of the most significant drawbacks of CCO is the inability to generate trees with asymmetric bifurcation ratios. In vascular systems, blood is transported over long distances inside bigger arteries, while only being in small arteries for a short distance. This leads to direct connections between small arteries and large trunks and to small bifurcation ratios. Only when approaching the smallest arteries can a shift to larger bifurcation ratios be observed. In contrast to these specific structures, CCO-generated trees tend to be more symmetric across all segments with flow evenly splitting into both branch segments. Many augmented versions of CCO were proposed to tackle this, often introducing post-processing steps and new constraints.

#### 2.2.2. Representative benchmark example

To summarize important characteristics of CCO-generated vascular trees and to establish a consistent way of quantifying them, we apply standard CCO to the benchmark problem introduced in Karch *et al.* [28]. The perfusion volume is a shallow rectangular box, and the root node is located at one of the corners. The model parameters are summarized in table 1.

**Table 1.** Model parameters of the benchmark problem due to Karch *et al.* [28]

parameter	meaning	value
$V_{\mathrm{perf}}$	perfusion volume	9 × 7 × 1.6 cm
$p_{\mathrm{perf}}$	perfusion pressure	100 mm Hg
$p_{\mathrm{term}}$	terminal pressure	60 mm Hg
$N_{\mathrm{term}}$	number of terminal segments	6000
$Q_{\mathrm{perf}}$	perfusion flow (at root)	500 ml min^−1^
η	blood viscosity	3.6 cP
γ	branching exponent	2.55
$N_{\mathrm{con}}$	maximum number of connections tested	{2, 4, 8, 16, 32, 64, 128}

**Figure 3.**
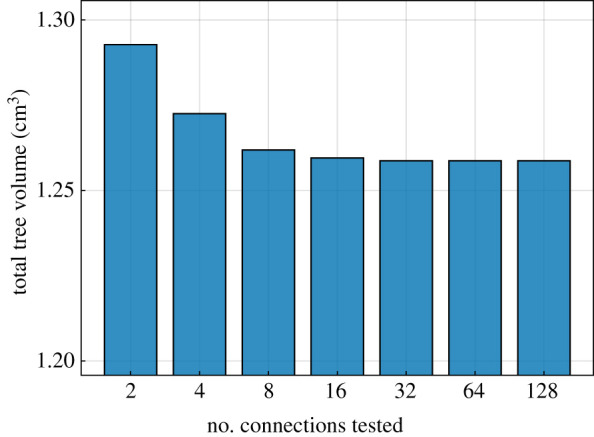
Total tree volume for different numbers of connection tests ($N_{\mathrm{term}}=6000$).

As stated above, CCO performs an optimization of the local tree structure. The topology optimization consists of connecting a newly sampled terminal node to different segments one after another. Only neighbouring segments are connected, and a maximum number $N_{\mathrm{con}}$ of connections is tested to make the computation more efficient. To determine an appropriate choice for $N_{\mathrm{con}}$ in our example, we generated seven trees with different values of $N_{\mathrm{con}}$, summarized in table 1. The total volumes of the resulting trees are compared in figure 3. Our results suggest a value of $N_{\mathrm{con}}=32$, as testing more connections had no significant influence on the final tree volume while increasing the overall computation time. We note that we will also use the value $N_{\mathrm{con}}=32$ for all further computations throughout the paper, including those in the context of our new framework that we will introduce below.

**Figure 4.**
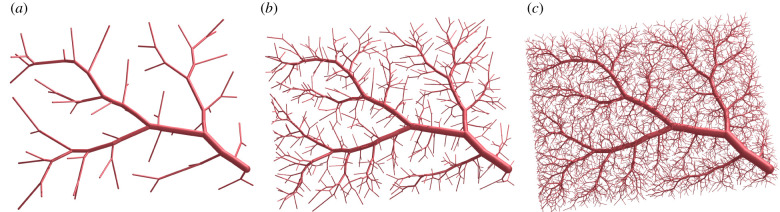
Different stages of a synthetic tree during CCO-driven growth. (*a*) $N_{\mathrm{term}}=50$, (*b*) $N_{\mathrm{term}}=500$ and (*c*) $N_{\mathrm{term}}=6000$.

Because of the iterative nature of the CCO approach, segments that are generated early on tend to define the overall hierarchy of the final tree. This phenomenon is illustrated in figure 4 for different numbers of terminal points. We observe that, after adding 50 terminal points only, the core structure is nearly identical to that of the final tree with 6000 terminal points. The reason for this is that CCO only changes the position of one bifurcation in each iteration. Therefore, positions of old bifurcations are fixed, and the corresponding segments do not change after initial generation. All previously optimized bifurcations, however, are no longer optimal after the next iteration. Furthermore, employing only subsequent disconnected geometry optimization steps tends to favour symmetric bifurcations, even for segments that appear further down the tree hierarchy.

**Figure 5.**
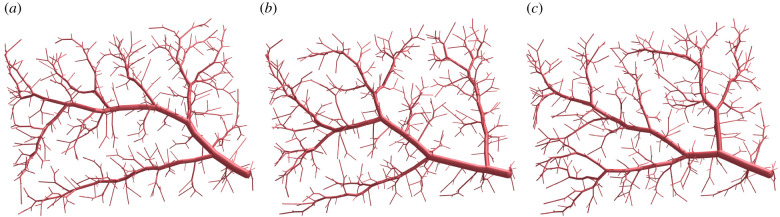
Different random seeds during CCO-driven growth ($N_{\mathrm{term}}=500$). (*a*) Seed 1, (*b*) seed 2 and (*c*) seed 3.

The bias of missing re-adjustments after adding new bifurcations is further amplified by the bias of the specific random seed on the initial sampling and the order in which samples are connected. This bias is illustrated in figure 5, where we used the same terminal points for each tree but connected them in the order defined by their random seed. We can observe that three different random seeds lead to three very different tree structures. Because of the dependence of the tree’s topology on the sampled terminal points, only qualitative comparisons are possible. A quantitative comparison of the exact segment locations against a real vascular system is not possible because results of the CCO method are not reproducible without pre-defining a fixed random seed.

### 2.3. A new approach based on optimizing the global geometry

The drawbacks of CCO are all due, at least partially, to optimizing only the local tree structure at each bifurcation. To mitigate this significant limitation and the associated problems, we introduce a new framework for generating synthetic trees that optimizes their geometry and topology. To this end, we formulate an NLP to optimize the *global* tree geometry, considering *all* branchings simultaneously. We furthermore add a heuristic step for the optimization of the tree topology. We cast these optimization steps into an algorithmic framework that uses CCO as a tool to grow the tree in between these optimization steps.

#### 2.3.1. Geometry optimization

We start with a CCO-generated tree T=(V,A) whose continuous variables serve as the initial estimate of the global geometry. We assume that we are given (for instance, via medical imaging) the root subtree of depth k with topology $T_{k}=(V_{k},A_{k})$, node locations ${\bar{x}}_{u}$, $u\in V_{k}$, as well as segment radii ${\bar{r}}_{a}$ and lengths ${\bar{\ell }}_{uv}=\|{\bar{x}}_{u}-{\bar{x}}_{v}\|$, $a=uv\in A_{k}$. If k = 0, only the root location ${\bar{x}}_{0}$ is provided. Locations ${\bar{x}}_{u}$ of all terminal nodes u∈L are given by sampling their spatial distribution. To circumvent the computationally expensive recursive computation of the radii $r={\left(r_{a}\right)}_{a\in A}$ as in Karch *et al.* [9] and similarly of the node pressures $p={\left(p_{u}\right)}_{u\in V}$, we include them together with the lengths $\ell ={\left(\ell_{a}\right)}_{a\in A}$ in the vector of optimization variables, *y* = (x, p, ℓ, r), where $x={\left(x_{u}\right)}_{u\in V}$. We have physical lower bounds $\ell^{-}$, $r^{-}$ on $\ell_{a}$, $r_{a}$, respectively, and we add artificial upper bounds $\ell^{+},\;r^{+}$ for numerical efficiency. Then y has to be an element of the box $Y=R^{4|V|}\times {\left[\ell^{-},\ell^{+}\right]}^{\left|A\right|}\times {\left[r^{-},r^{+}\right]}^{\left|A\right|}$ of dimension 4|V|+2|A|=6|V|−2, and our NLP reads:

2.7 $$\underset{y\in Y}{\min}\;\sum_{a\in A}\ell_{a}r_{a}^{2},$$

2.8 $$\text{s.t.}\;\;0=x_{u}-{\bar{x}}_{u},\;u\in V_{k}\cup L,$$

2.9 $$0=\ell_{a}-{\bar{\ell }}_{a},\;a\in A_{k},$$

2.10 $$0=r_{a}-{\bar{r}}_{a},\;a\in A_{k},$$

2.11 $$0=\ell_{uv}^{2}-\|x_{u}-x_{v}{\|}^{2},\;uv\in A\setminus A_{k},$$

2.12 $$0=r_{uv}^{\gamma }-\sum_{vw\in A}r_{vw}^{\gamma },\;v\in V\setminus (V_{k}\cup L),$$

2.13 $$0=p_{u}-p_{v}-\left(\frac{8\eta }{\pi }\right)Q_{uv}\ell_{uv}/r_{uv}^{4},\;uv\in A,$$

2.14 $$0=p_{u},\;u\in L,$$

and

2.15 $$0=p_{0}-\Delta_{p}.$$

Here, (2.8)–(2.10) fix the geometry of the root tree $T_{k}$ and the locations of all terminal nodes. Constraints (2.11) and (2.12) ensure consistency of $\ell_{uv}$ with *x*_*u*_, *x*_*v*_ and Murray’s law, respectively, outside $T_{k}$. The pressure drops across segment uv and the terminal pressure $p_{u}$ are given by (2.13) and (2.14), respectively, where $Q_{uv}=\sum_{vw\in A}Q_{vw}$ for v∈V∖({0}∪L) (Kirchoff’s law) and $Q_{uv}=Q_{\mathrm{perf}}/\left|L\right|$ for v∈L (homogeneous flow distribution). Constraint (2.15) fixes the pressure drop from the root node to the terminal nodes at the prescribed value Δ_*p*_. Moreover, we set $p_{\mathrm{term}}=0$ without loss of generality.

We use lower bounds *r*^−^ = 0.1 mm, the radius of vessels entering the microcirculatory network [8], and $\ell^{-}=0.2\;\mathrm{mm}$ to satisfy the conditions for Poiseuille flow to hold also for the smallest vessels. The upper bounds are $\ell^{+}=2\max_{a\in A}\ell_{a}^{\mathrm{in}}$ and $r^{+}=2\max_{a\in A}r_{a}^{\mathrm{in}}$, where $\ell_{a}^{\mathrm{in}},r_{a}^{\mathrm{in}}$ refer to the initial CCO-generated tree. If the length of a non-terminal segment becomes smaller than its diameter we delete it. We then replace this degenerate segment with its branch segment, which may create a trifurcation.

**Figure 6.**
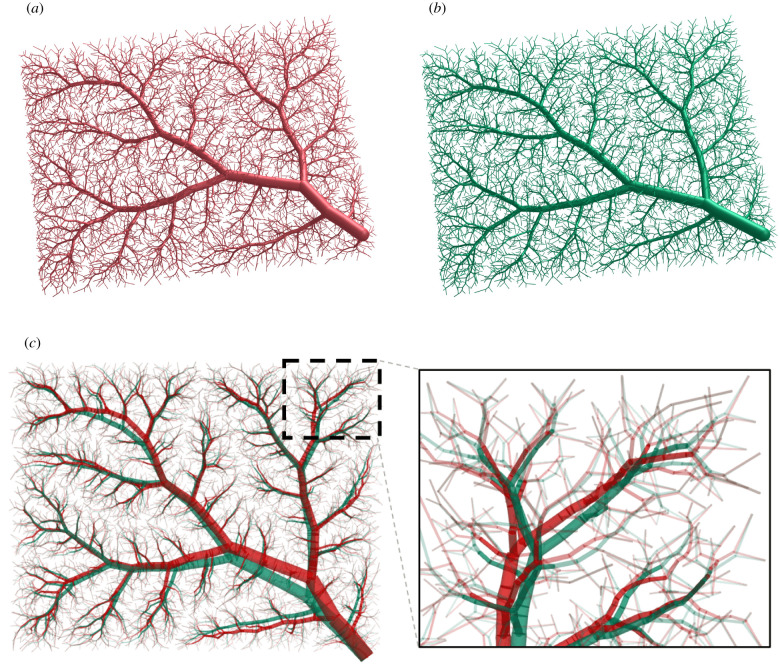
Comparison of complete tree structures (with $N_{\mathrm{term}}=6000$). (*a*) Standard CCO-driven growth, (*b*) CCO + geometry optimization via NLP and (*c*) overlaid geometries (red = standard CCO, green = CCO + geometry optimization).

We use our benchmark problem due to Karch *et al.* [28] with $N_{\mathrm{term}}=6000$ terminal segments to assess the effect of geometry optimization via the NLP described above. To this end, we first compare visualizations of the complete tree structure generated via standard CCO in figure 6*a* and geometrically optimized afterwards by solving the NLP in figure 6*b*. We overlay the geometries of both trees in figure 6*c* and observe that, although at this stage the tree topology remains the same, the two methods lead to significant differences in tree geometry. As a result of the NLP, the total volume of the tree is reduced by 4.1% compared with the standard CCO tree. Furthermore, 566 trifurcations are created during the optimization.

As a measure of the symmetry between branches, the *branching ratio* of node *u* is defined as [8]

2.16 $$\delta_{u}=\frac{\text{min}\{r_{uv}:\;uv\in A\}}{\max\{r_{uv}:\;uv\in A\}}\;\forall u\in V\setminus L.$$

To show the impact of repeated geometry optimizations on the overall branching asymmetry, we compute the branching ratios (2.16) over all generations.

**Remark 2.1.**

To classify the hierarchy throughout the tree, each segment is assigned to a generation according to the Strahler ordering method [29]. The ordering starts from the leaf nodes, which are initially assigned to the order 1. At each branching, the parent node is assigned the maximum order of its children. If the children belong to the same order, the parent is assigned the order of its children plus 1. For each generation, the Strahler order is applied contrariwise, starting with the root segment at generation 1.

**Figure 7.**
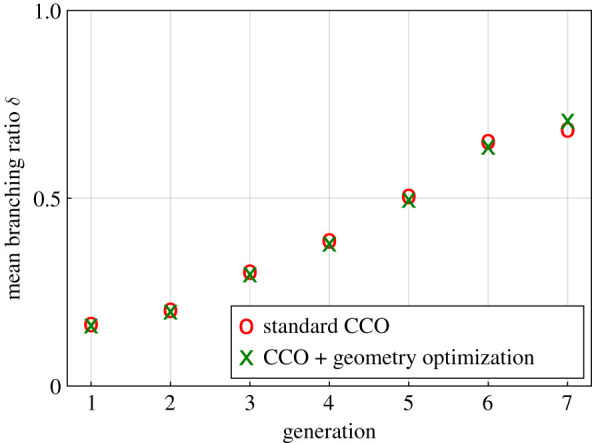
Comparison of branching ratios between a CCO-generated tree and a geometrically optimized tree.

Figure 7 plots the branching ratios for the first seven generations for the geometrically optimized tree and the standard CCO-generated tree. We observe that optimizing the *global* geometry improves the branching asymmetry over generations 2–6. We note that, for higher generations, branching ratios of both trees become more symmetric. This is consistent with observations in corrosion casts [4], where smaller vessels also tend to bifurcate more symmetrically.

**Figure 8.**
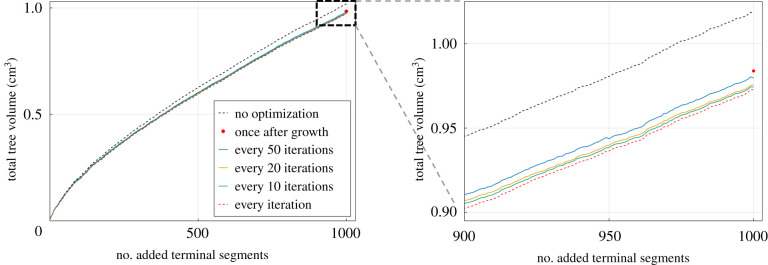
Influence of the number of geometry optimizations during growth on the final tree volume ($N_{\mathrm{term}}=1000$).

Optimizing the global geometry repeatedly during the branching process proves beneficial, especially for very large trees, but doing this each time after a node is added is computationally expensive. To reduce the associated computational cost, our idea is to run this optimization after several nodes are added. To determine an appropriate rule that balances accuracy and computational cost, we conduct a sensitivity study for the current benchmark problem with $N_{\mathrm{term}}=1000$. Based on the results of this study (shown in figure 8), we find that carrying out geometry optimization after $N_{\mathrm{geo}}=20$ new nodes is an appropriate compromise for sparser trees, which we will increase step-wise during growth to a maximum of 500 for the densest trees (more than 20 000 nodes). We will apply this rule in all computations in the remainder of this paper.

#### 2.3.2. Topology optimization

We have seen that optimizing the global geometry reduces the total volume of the vascular tree and improves its asymmetric branching pattern. However, the locations of nodes still depend primarily on the sampling of the terminal points in the CCO algorithm. To reduce the associated bias, we propose an additional topology optimization step for an intermediate tree structure with fewer total segments. We use the property of CCO that initial samples are not changed significantly during growth by continuing the growth from this intermediate vascular structure.

We optimize the topology by exchanging pairs of proximal points from one parent segment to another and then optimizing the global geometry using the NLP model. This is similar to the local search for the best connection in the standard CCO algorithm, with the key property of also allowing the swapping of entire subtrees. Our topology optimization approach is discrete, and the total number of possible topologies for a binary branching tree with n nodes is given by the Catalan number,

2.17 $$C_{n}=\frac{1}{n+1}\left(\begin{array}{c} 2n\\ n \end{array}\right).$$

For only $N_{\mathrm{term}}=500$ segments this still involves 50 000 possible swaps per iteration. To reduce this number, we delete infeasible swaps that create a cycle (an ancestor node is connected to the current node) and swaps where the initial new segment length is at least two times larger than the current segment length. During tests, we observed that these swaps almost never lead to improved topologies. Since the root subtree $T_{k}$ and the leaf locations are kept fixed, this restricts the search to local topology changes. The number of possible swaps per iteration then drops to around 7500.

This number is still too large to search the entire possible solution space. We therefore deploy SA [30], a metaheuristic approach, to search the discrete solution space. Instead of accepting a new topology only when it yields a smaller volume than the current one, SA accepts worse topologies with a probability of

**Figure 9.**
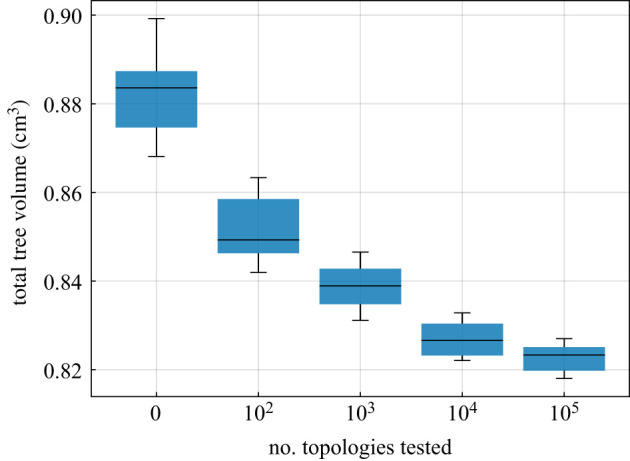
Box plot of the total volume of 10 trees generated with different seeds obtained for different numbers of tested topologies during discrete topology optimization ($N_{\mathrm{term}}=500$).

2.18 $$p=\exp\left(-\frac{\Delta f_{T}}{T}\right),$$

where $\Delta f_{T}=f_{T}^{j}-f_{T}^{i}$ is the change in cost associated with going from topology i to topology j. T is the SA temperature, which is ‘large’ initially and is then ‘cooled down’ after each iteration. This means that SA can ‘climb out’ of local minima and search a wider solution space. Figure 9 shows the total volume of 10 different trees during topology optimization with SA in a box plot, illustrating the effectiveness of the approach. We observe that not only the topology optimization significantly reduces the total volume but also the variance between different trees is reduced. This indicates that the different random seeds converge to nearly identical tree structures.

Since we use CCO to obtain the initial tree topology, the initial temperature $T_{0}$ does not need to be chosen too large, which significantly reduces computation time.

#### 2.3.3. Combining geometry and topology optimization

To complete our new optimization framework, we combine geometry and topology optimization. We specify the perfusion volume to be filled, the number of terminal segments $N_{\mathrm{term}}$ and the initial root subtree of depth k (or proximal point of root for k = 0). After initialization of the problem, we use CCO to grow the tree until it has 500 terminal segments. We optimize the topology of this initial tree, as described in §2.3.2. From this near-optimal tree, we restart CCO until $N_{\mathrm{term}}$ segments are added. After each $N_{\mathrm{geo}}$ iteration step, we optimize the global tree geometry by solving the NLP. The structure of our framework is shown in algorithm 1.


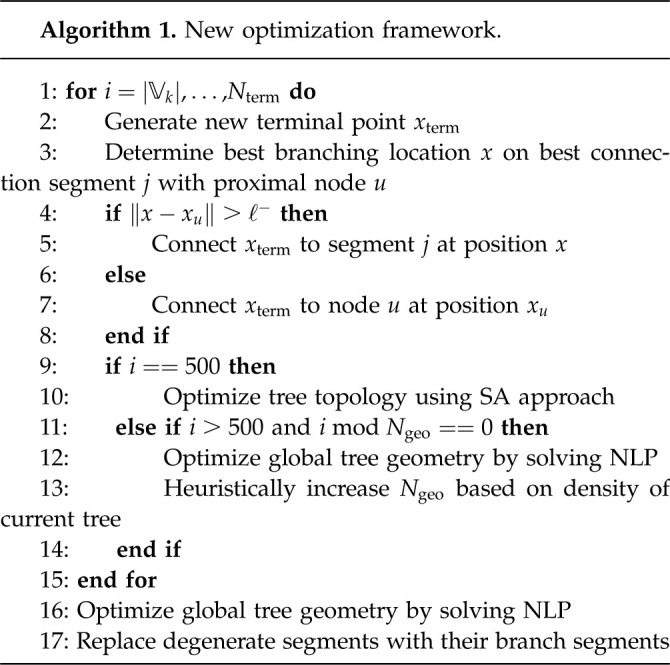


Our new optimization framework together with the CCO algorithm was implemented in the programming language *Julia* [31] and will be the basis for further studies of the perfusion behaviour inside the liver. We plan to make the entire framework open access in the next 2 years. The NLP is solved by an interior point method using the solver *Ipopt* [32] and the linear solver *Mumps* [33].

All computations were done on a desktop computer with 32 GB of random-access memory (RAM) and an Intel Core i9-9900k @5Ghz with 16 processing threads.

To measure the computation cost of each component of our framework, we measured the computing times for three different cases. The first two cases include the benchmark problem with $N_{\mathrm{term}}=500$ and $N_{\mathrm{term}}=6000,$ respectively, and the third is the generation of a portal vein (PV), described in §3, with $N_{\mathrm{term}}=24\;000$. We formally divide our framework into CCO-driven growth, geometry optimization during growth and topology optimization on the reduced tree ($N_{\mathrm{term}}=500$). The results are shown in table 2. It becomes clear that (except for $N_{\mathrm{term}}=24\;000$) the topology optimization using the SA approach is the most expensive part of the framework, even though we are limiting it to only 500 terminal segments. By contrast, optimizing the global geometry during growth is efficient even for the PV problem. It takes 45 s to solve the NLP for 24 000 terminal segments.

**Table 2.** Computing times of the new optimization framework for three different cases.

	benchmark		portal vein
	($N_{\mathrm{term}}=500$)	($N_{\mathrm{term}}=6000$)	($N_{\mathrm{term}}=24\;000$)
CCO-driven growth	10 s	565 s	8640 s
geometry optimization	15 s	285 s	2930 s
topology optimization	4820 s	4970 s	6126 s

**Figure 10.**
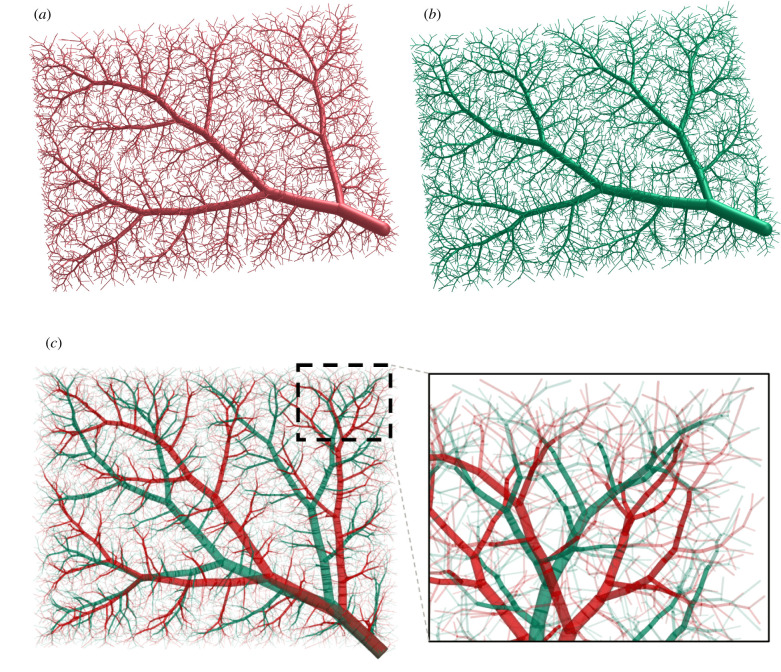
Vascular trees before and after topology optimization ($N_{\mathrm{term}}=6000$). (*a*) Geometry optimization only (via series of NLPs), (*b*) geometry and topology optimization (via series of NLPs + discrete topology testing) and (*c*) overlaid geometries (red = geometry optimization only, green = geometry and topology optimization).

Using our current benchmark example, figure 10 enables a visual comparison of the complete vascular tree that is geometrically optimized via solving the NLP and the complete vascular tree that is geometrically and topologically optimized. We observe that the geometrically and topologically optimized tree differs significantly from the tree that is only geometrically optimized.

**Figure 11.**
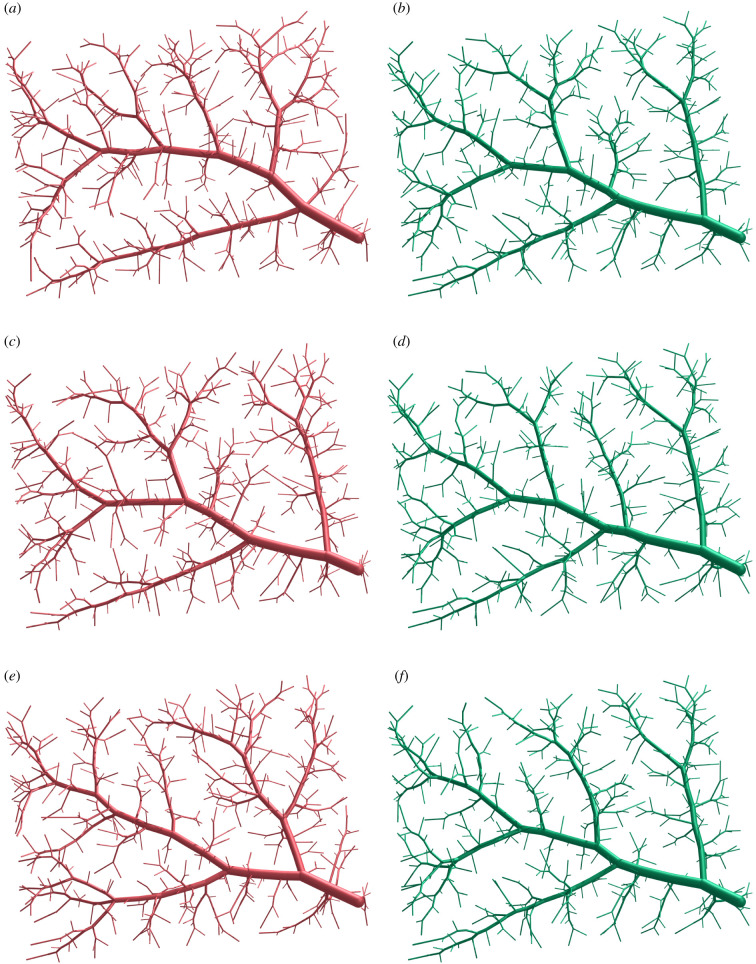
Trees generated with different random seeds before and after topology optimization ($N_{\mathrm{term}}=500$). (*a*) Seed 1 (geometrically optimized), (*b*) seed 1 (geometrically + topologically optimized), (*c*) seed 2 (geometrically optimized), (*d*) seed 2 (geometrically + topologically optimized), (*e*) seed 3 (geometrically optimized) and (*f*) seed 3 (geometrically + topologically optimized).

To better illustrate the importance of topology optimization, we consider the three geometrically optimized trees with $N_{\mathrm{term}}=500$ that are shown in the left column of figure 11. They are juxtaposed to the corresponding versions after having applied the topology optimization.

**Figure 12.**
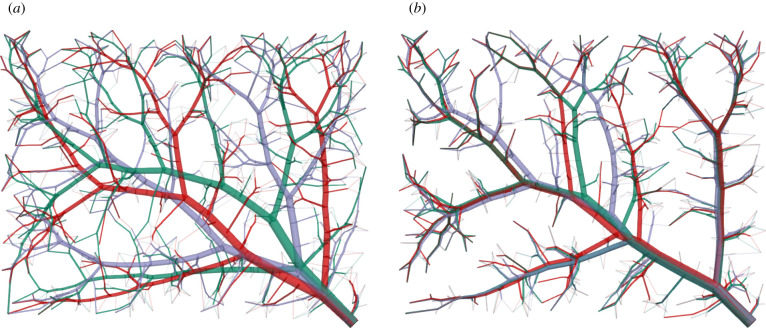
Overlaid geometries of different random seeds before and after topology optimization ($N_{\mathrm{term}}=500$; green = seed 1, red = seed 2, purple = seed 3). (*a*) Seeds (geometrically optimized) and (*b*) seeds (geometrically + topologically optimized).

For the current benchmark, topology optimization further reduces the total volume of the tree by up to 6%, resulting in a total volume decrease of up to 11% with respect to the standard CCO-generated trees. We can also observe in figure 11 that all three trees, although generated with different random seeds, converge towards very similar tree structures. This convergence is also highlighted in figure 12, where we overlaid the different trees before and after topology optimization, respectively. In particular, we see in all three results a prominent large trunk going from the bottom right corner to the top left corner, connecting two main branches on the top side and one main branch on the bottom side.

## 3. Validation

**Figure 13.**
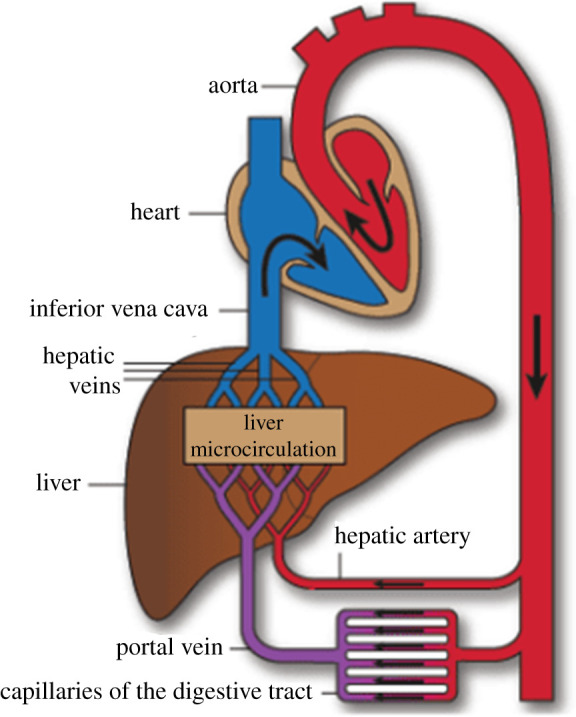
Schematic overview of the liver inside the systemic circulation; from Debbaut [34].

We have developed a framework based on mathematical optimization that allows us to generate synthetic vascular trees with reproducible topology and geometry for general non-convex perfusion volumes. We can now validate the overall approach against real vascular systems. To this end, we consider the hepatic vascular systems in the human liver. Blood flow through the liver on the organism scale is shown in figure 13. In contrast to other organs, the liver has two supplying trees. The first one is supplied through the *hepatic artery* (HA) from the heart, and the second one is supplied through the PV from the digestive tract. The blood leaves the liver through a single draining tree into the *hepatic veins* (HVs), leading into the inferior vena cava (IVC).

In the scope of this work, we focus on the supplying tree that stems from the PV. We apply our framework for generating a synthetic hepatic tree that we can then assess via a real hepatic tree that is experimentally characterized via a detailed vascular corrosion cast of a human liver.

### 3.1. Vascular corrosion casting

As *in vivo* medical imaging cannot provide detailed representations of hepatic tree structures, we resort to *ex vivo* vascular corrosion casting, as described in detail by Debbaut *et al.* [4]. We compare the synthetic trees against a human liver previously discarded for transplantation owing to failed reallocation. The protocol conforms to the ethical guidelines of the 1975 Declaration of Helsinki, and was approved by the ethics committee of the University Hospitals Leuven, Belgium, and by the Belgian Liver and Intestine Committee as foreseen by the initial protocol, as stated in Debbaut *et al.* [4]. The *ex vivo* liver (weight ≈1.9 kg) was first connected to a machine perfusion preservation device (Organ Recovery Systems, Zaventem, Belgium). During a 24 h period, the liver was continuously perfused under pressure control through the HA at 25 mm Hg and the PV at 7 mm Hg. The blood left the liver through the HVs and IVC. Perfusion of the liver allows the preservation of the vasculature and parenchyma. Moreover, it keeps the blood vessels open. The colour-dyed casting resin was added to both the HA and PV simultaneously until a sufficient quantity emerged from the IVC. Afterwards, inlet and outlet vessels were clamped to avoid resin leakage during the polymerization step, which took approximately 2 h. After 2 days in a macerating bath, the corrosion cast was ready for imaging. The liver cast was imaged *in globo* and the resulting image dataset was reconstructed using Octopus software (Ghent University, Ghent, Belgium). Imaging was done using a high-resolution micro-CT scanner. The whole cast was imaged at a resolution of 102 μm, recovering vessels down to a diameter of approximately 0.5 mm. Because of labour intensity reasons, a second analysis was carried out only on a smaller subsample (about 88 × 68 × 80 mm) at a resolution of 71 μm. For this sample, vessels down to a diameter of approximately 0.08 mm were recovered for later processing. The complete casting and micro-CT set-up is illustrated in [4]. More detailed information on the vascular corrosion casting and micro-CT scanning can be found in [35].

**Figure 14.**
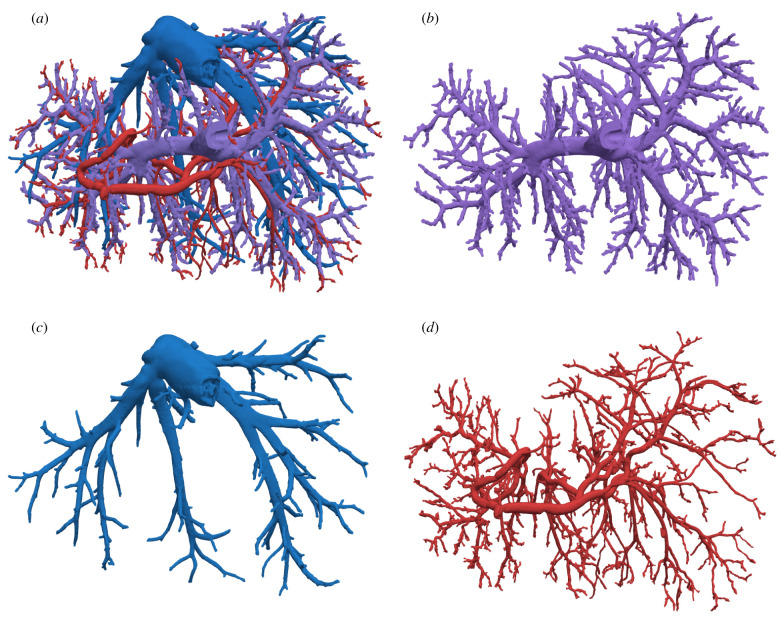
Representations of all three vascular hepatic trees obtained from imaging of the corrosion cast as obtained in [4]. (*a*) Segmented image of all three vascular trees, (*b*) segmented image of the PV, (*c*) segmented image of the HVs and (*d*) segmented image of the HA.

The resulting micro-CT dataset was processed and segmented based on the grey values of the images. The separation of arterial and venous vessels was facilitated by the contrast agent used in the HA resin. The separation of the PV and HV trees, however, was more challenging because of similar grey values and touching vessels, requiring manual segmentation. After the segmentation, a three-dimensional reconstruction of each tree was calculated. The resulting geometries are shown in figure 14.

A detailed visual inspection of the tree representations shows that, in addition to bifurcations, all trees also exhibit a number of trifurcations. We also observe monopodial branches sprouting from parent vessels at angles close to $90^{\circ }$. After the first generations, the HA vessels typically run parallel to the PV vessels. This trend continues down to the microcirculation. From the macro- to the mesocirculation, the mean radii decreased to 0.08 mm at the most distal mesocirculation generation 13 in the sample studied in [4]. At the microcirculation level, blood reaches the functional units of the liver, called hepatic lobules. This smallest scale of the circulation exhibits entirely different flow characteristics [19] that we cannot describe with our model. Instead, more specific models as in [36] would be needed.

### 3.2. Comparison and assessment

The synthetic generation of the PV tree is based on the perfusion volume of the experimentally investigated tree from Debbaut *et al.* [4] and the physiological parameters taken from Kretowski *et al.* [37]; see table 3. We generate the vascular tree with $N_{\mathrm{term}}=24\;000$ segments, both with the standard CCO method and with our new framework as described in §2.3.3. Our framework takes 4 h 50 min, while the standard CCO method takes 2 h 37 min; see table 2.

**Table 3.** Physiological parameters required for the generation of a hepatic vascular tree (portal vein); adapted from Kretowski *et al.* [37]

parameter	meaning	value
$V_{\mathrm{perf}}$	perfusion volume	≈1500 cm^3^
$p_{\mathrm{perf}}$	perfusion pressure	12 mm Hg
$p_{\mathrm{term}}$	terminal pressure	8 mm Hg
$N_{\mathrm{term}}$	number of terminal segments	6000
$Q_{\mathrm{perf}}$	perfusion flow (at root)	1000 ml min^−1^
η	blood viscosity	3.6 cP
γ	branching exponent	3.0
$N_{\mathrm{con}}$	number of connections tested	30

**Figure 15.**
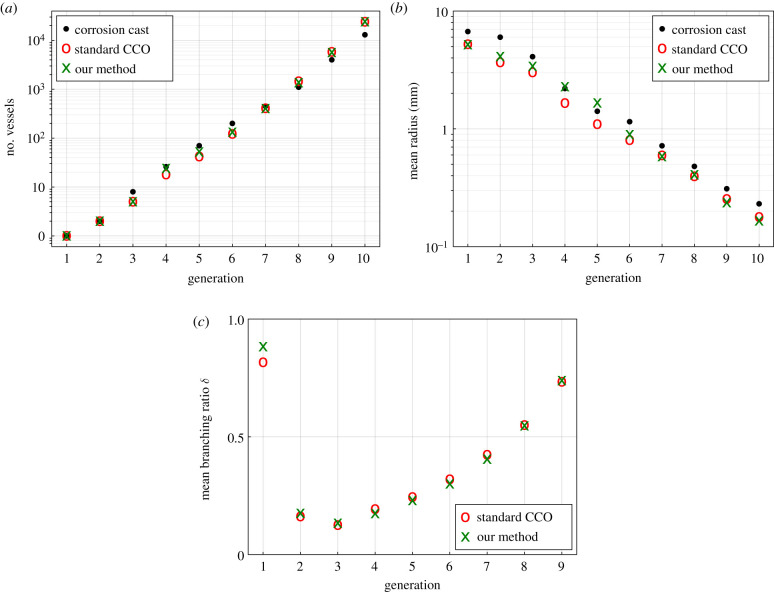
Key statistics of the portal vein tree: our method versus CCO and corrosion cast measurements. (*a*) Number of vessels, (*b*) mean radii and (*c*) mean branching ratios.

We start the analysis by a qualitative comparison of the segment parameters averaged over each generation. In figure 15, we compare the number of segments and segment radii between standard CCO, our method based on optimizing the global geometry and the reference values calculated by Debbaut *et al.* [4] based on corrosion cast measurements, for each generation of the hierarchical tree structure. We can observe in figure 15*a* that the number of vessels per generation deviates only slightly between all three cases. Comparing the average radius per generation in figure 15*b*, however, indicates that our method fits the corrosion cast data better than the standard CCO results for the important lower generations between 1 and 6. Optimizing the global geometry shortens the overall segment length of the intermediate generations, leading to larger radii overall. By contrast, CCO overestimates the lengths in these generations owing to the limiting view of optimizing the local geometry only, which leads to smaller radii overall. For higher generations beyond 7, both methods seem to underestimate the corrosion cast data. However, we note that the choice of the branching exponent γ significantly influences the values of the vessel radii, limiting the possible improvement due to the optimization framework. The improvement in the branching asymmetries in figure 15*c* is also significant, especially for generations 4–6. The high branching ratio for generation 1 signifies that the root segment branches symmetrically into daughter branches with similar radius. The branching asymmetries for the lower generations increased, leading to an increase in monopodial branches and an overall higher number of thicker vessels, which is also visually more comparable to the corrosion cast. The branching ratios tend to be larger for the higher generations and are comparable for both the standard CCO and our method. This is also supported by Debbaut *et al*. [4] in the corrosion cast of a smaller mesoscale sample.

**Figure 16.**
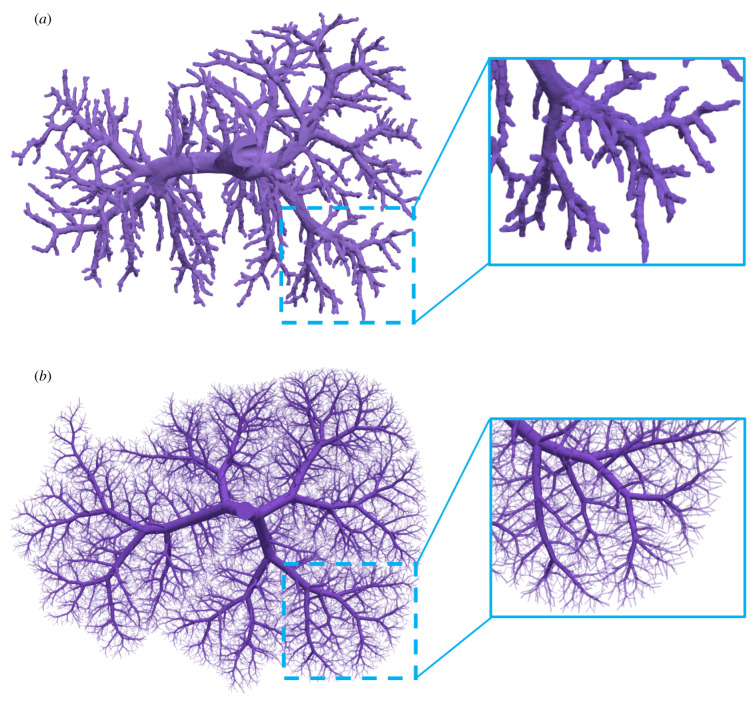
The complete portal vein tree: synthetically generated tree structure versus corrosion cast data. (*a*) Corrosion cast and (*b*) our method.

Finally, figure 16 shows the synthetically generated tree structure of the PV and the corrosion cast data below each other. In addition to bifurcations, the corrosion cast data exhibit 34 trifurcations. In our method, the tree exhibits 41 trifurcations over the first seven generations, whereas in standard CCO trifurcations are impossible by design. Furthermore, the number of monopodial branches increased from 341 to 521 from the standard CCO tree to our tree. Lastly, the visual comparison of the synthetic tree structure based on optimizing the global geometry with the corrosion cast data shows good agreement, especially for the early generations. In particular, in both trees, the root vessels split horizontally (with respect to the depicted view) and have seven major arteries (generations 2 and 3) splitting from there. Zooming in to the bottom right corner, we observe that both trees show highly similar branching patterns. We also see, however, that, in other areas, there are pronounced differences. For instance, the bottom centre of the synthetic tree is supplied uniformly via a larger vessel that diagonally stretches downwards, whereas the corresponding area in the corrosion cast is nearly empty. Also, the overall distribution of the radii of the vessles is qualitatively different, as was already indicated in figure 15*b*. However, we want to highlight that the influence of different values for the branching exponent γ had only a limited impact on the overall topology and vessel positions.

## 4. Discussion and outlook

The core assumption behind the synthetic generation of vascular trees is that their physiological formation is governed by optimality principles to reduce the overall metabolic demand. Current synthetic tree generation methods such as CCO are capable of reproducing qualitative measures of their real counterparts, but fail to achieve comparable branching patterns. Furthermore, owing to dependence on random sequences, methods such as CCO cannot guarantee reproducibility of their results, making a quantitative comparison and validation nearly impossible. We showed that these drawbacks also stem from the fact that standard methods such as CCO are based only on optimizing the local tree structure.

In this paper, we developed a new powerful framework for generating synthetic vascular trees to mitigate the above limitations. The fundamental basis of our framework is the search for a minimum in both the tree’s global geometry and global topology. In contrast to standard methods, we split this search into a distinct geometry optimization and a topology optimization. This allows us to formulate the geometry optimization as an NLP. Unlike other methods, this permits efficient solution algorithms such as the interior point method, vastly improving the overall computation time. We combine CCO with a subtree-swapping procedure for the topology optimization to search between different topologies iteratively. In each iteration, we optimize the geometry of the new topology by solving the NLP. We use a metaheuristic algorithm, similar to SA, to either accept or reject a new topology. Finally, we combine these steps into a single algorithmic approach.

Our new algorithm is capable of generating synthetic trees with up to 11 generations. As input, we only need the (non-convex) volume that is perfused and the root segment’s entry point. The resulting trees showed improved branching patterns while reducing the metabolic cost by up to 11%. Furthermore, results are reproducible, and the influence of random seeds on the global structure is significantly reduced. This allowed us to directly compare a synthetic hepatic tree against the PV of a liver corrosion cast. Our comparison showed similar branching patterns and comparable geometric locations of both the segments and branchings. In areas where the influence of the geometry of the HVs is not strong, these similarities reach down to the fifth generation. Also, the number of trifurcations and monopodial branches formed during growth is close to that of the real hepatic tree characterized by the corrosion cast data.

The direct comparison with the corrosion cast data also showed some limitations of the current framework that we would like to address in future work. Formally, we can categorize these into model related, application (liver) related and method related. On the model part, we made significant assumptions, namely for the blood viscosity and the cost function. The blood viscosity should take the Fåhræus–Lindqvist effect into account. The cost function only considers the total volume as the minimization goal. In addition, the assumption of a constant value of a branching exponent γ needs to re-evaluated. During all tests, the influence of γ on the radii of the vessels was significant. Lastly, further factors such as the transport cost of blood should be considered as additional optimization goals. The results for the liver application highlighted key areas where the synthetic tree deviated significantly from the corrosion cast (mainly at the bottom centre). We hypothesize that this deviation is due to the missing HVs and HA that are not considered in the synthetic model but are present in the corrosion cast; see figure 14. This region is also close to where the gallbladder is typically situated. This will be similar in other organs with clearly defined inflow and outflow trees. As such, our framework should be extended to allow the generation of both trees in a coupled manner. All these extensions of the framework will certainly increase the overall computational complexity. This means that the method’s efficiency must be further improved. Currently, using the NLP model for geometry optimization is both robust and efficient, and CCO combined with the heuristic subtree-swapping procedure is a good practical approach for searching the discrete space. However, a proper mixed-integer nonlinear optimization model (MINLP) for topology optimization would be desirable. Such a MINLP model provides a rigorous formulation of the combined topology and geometry optimization, and it is necessary if we wish to replace heuristic topology optimization approaches such as SA by rigorous mathematical methods such as branch-and-bound algorithms. Although solving the MINLP is extremely hard and would require a substantial mathematical research effort, it might ultimately produce better topologies, and it could even provide optimality certificates for the solutions.

We expect that our framework could be helpful in several applications. First, synthetic trees generated by this framework could help to improve the interpretation of medical images by, for example, artificially increasing the density of the initial segmented tree to the desired pre-arteriolar level. Such dense trees could give valuable input to the functional assessment of organs such as the liver [38] and the heart [39]. One primary application domain would be the treatment of cancer. Synthetic trees could compute the blood flow towards the tumour, supporting various clinical decisions, e.g. the dosage of chemotherapeutic agents. Another example would be liver resection, where part of the liver containing the tumour is surgically removed [40]. Here, synthetic trees could help identify optimal cut patterns, which reduce the risk of liver failure while increasing the probability of cutting away the complete tumour.

## Data Availability

Data presented in this article can be found in three compressed zip folders as the electronic supplementary material [41]. The first folder includes all generated trees in VTK-file format, capable of three-dimensional inspection. The second folder consists of all trees in POV-file format, capable of raytracing and plotting the data. The third folder includes all trees in Julia-file format, capable of plotting different statistical values for each tree.
